# Continuous Murmur in a Child: Sometimes It Is a Zebra

**DOI:** 10.1016/j.case.2023.02.006

**Published:** 2023-04-18

**Authors:** Edward H. Hardison, Sudeep D. Sunthankar, Joshua D. Chew, Jeffrey G. Weiner

**Affiliations:** a Department of Internal Medicine and Pediatrics, Vanderbilt University Medical Center, Nashville, Tennessee; b Division of Pediatric Cardiology, Department of Pediatrics, Monroe Carrell Jr. Children's Hospital at Vanderbilt, Nashville, Tennessee

**Keywords:** Arteriovenous fistula, Continuous murmur, Congenital, Aorta, Innominate vein

## Abstract

• Most AVFs present with a continuous murmur with radiation to the back.
• There is little evidence to guide management of thoracic AVF.
• Management options include surgical repair, embolization, or conservative management.
• Conservative management is a reasonable approach in asymptomatic patients.

## Graphical abstract

**Figure:**
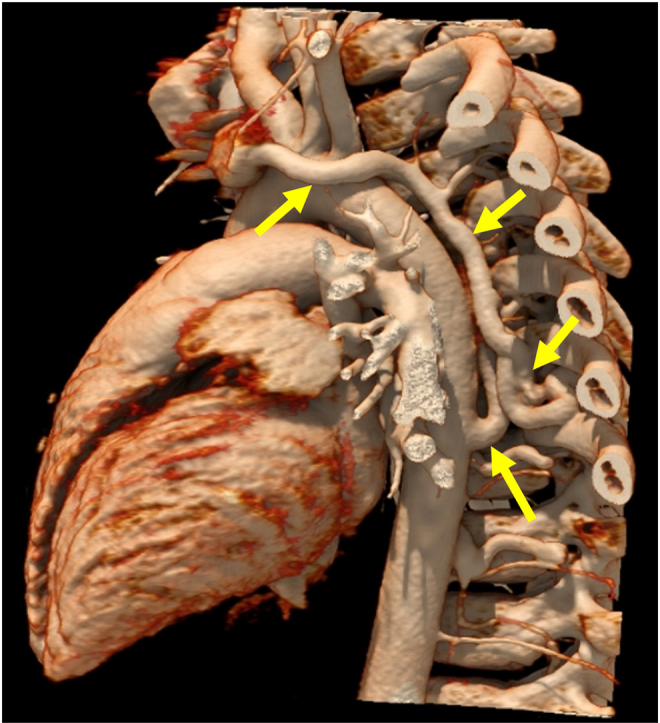

## Introduction

Auscultation of a heart murmur is a common reason for concern and referral to a pediatric cardiologist. Distinguishing a benign from a high-risk murmur requires careful characterization and an experienced examiner. We describe an asymptomatic pediatric patient who presented with a 3/6 harsh, blowing, continuous murmur audible along the left posterior scapular border. Our objectives in this report are to (1) elucidate the causes of a continuous murmur, (2) briefly describe the congenital and acquired causes of thoracic arteriovenous fistulae (AVFs), and (3) discuss our rationale for conservative management of our patient.

## Case Presentation

**Figure 1:**
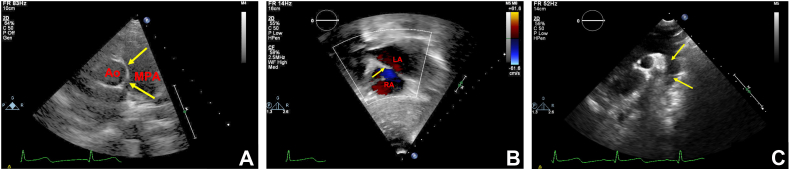
**(A)** Two-dimensional TTE (5 MHz transducer) high right parasternal view demonstrates intact septation between the aorta and MPA *(arrows)*. **(B)** Subcostal coronal view with color flow Doppler demonstrates the intact atrial septum *(arrow)*. **(C)** Suprasternal sagittal view demonstrates a widely patent aortic arch *(arrows)*. *LA*, Left atrium; *MPA*, main pulmonary artery; *RA*, right atrium.

**Figure 2:**
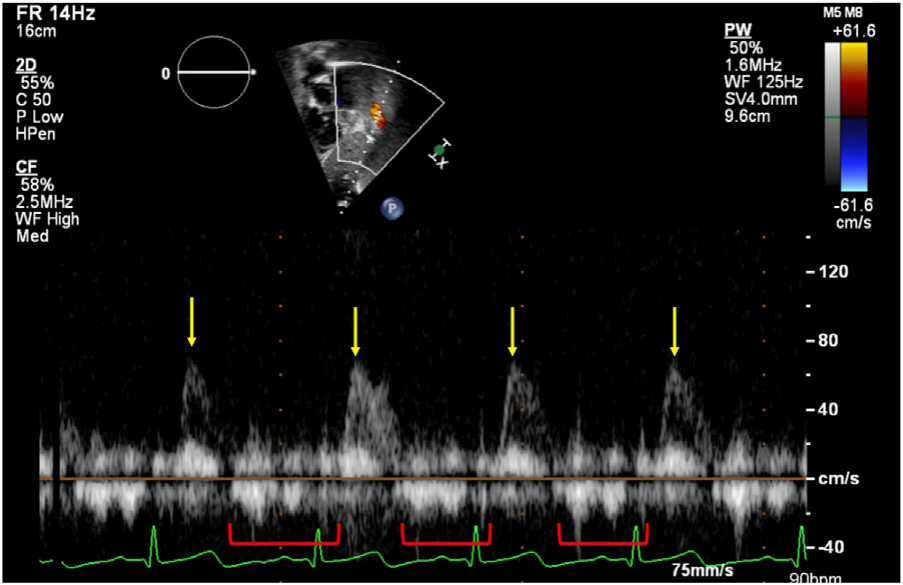
Two-dimensional TTE subcostal sagittal view with color flow–guided pulsed-wave Doppler spectrum demonstrates a pulsatile abdominal aortic Doppler *(arrows)* pattern without a diastolic runoff pattern *(red bracket)* at this angle of interrogation.

**Figure 3:**
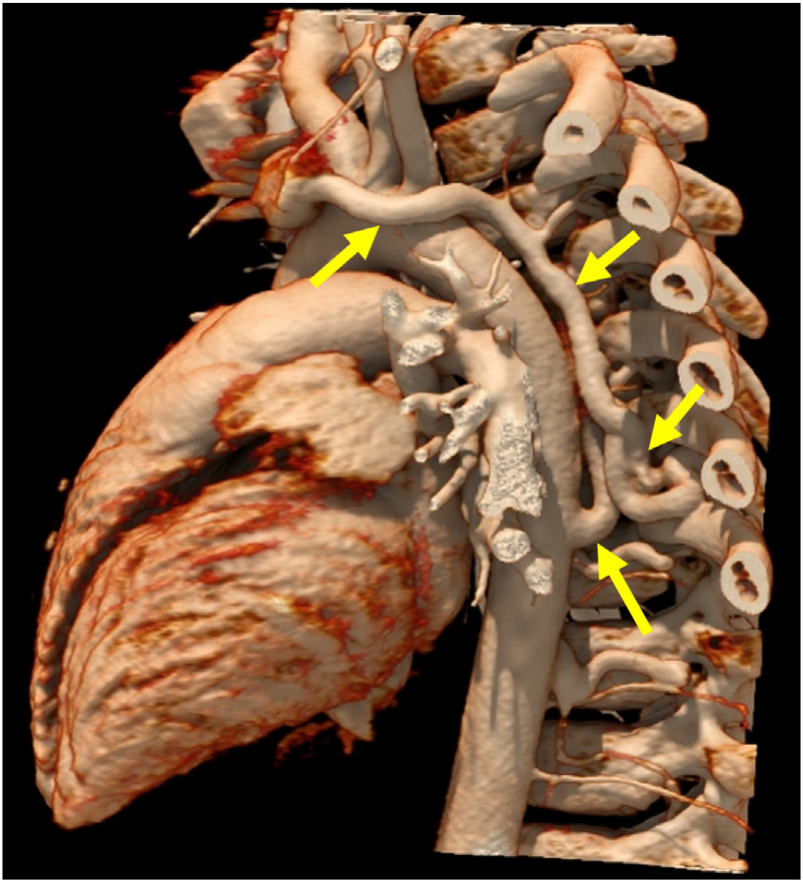
Chest CTA, three-dimensional volume-rendered reconstruction demonstrates an abnormal vessel *(arrow)* arising from the posterior aspect of the descending thoracic aorta at T7-T8 and extending superiorly in a tortuous manner along the posterior mediastinum. This vessel provides several branches to the epidural plexus and eventually connects to the left innominate vein *(arrow)*.

A 9-year-old child was referred to the cardiology clinic for a heart murmur. Clinically, our patient reported being asymptomatic with the ability to participate in all childhood activities without limitation. Electrocardiogram showed prominent Q waves in the inferior leads. Auscultation demonstrated a 3/6 harsh, blowing, continuous murmur audible along the left posterior scapular border without radiation. No murmur was audible with anterior chest wall auscultation. The patient had normal blood pressure measurement with a normal pulse pressure. Transthoracic echocardiogram (TTE) was obtained due to concern for aortic disease, such as patent ductus arteriosus, aortopulmonary window, or aortic dissection; however, the inability to auscultate a murmur from the anterior chest wall was puzzling. Aortic dissection was less likely given that the patient appeared well. Echocardiogram showed normal chamber size (left ventricular internal dimension in diastole = 41 mm, *Z* score = 0.00; left ventricular end-diastolic volume = 88.0 mL, *Z* = 1.2), normal segmental relationships, no intracardiac shunting, normal valve annuli sizes, normal valvular velocities, and no valvular dysfunction (Video 1, Figure 1). Color and spectral flow Doppler of the abdominal aorta demonstrated no retrograde diastolic flow or runoff (Figure 2). Due to the quality of the murmur despite a normal TTE, a chest computed tomographic angiogram (CTA) was completed to investigate other arterial malformations. This identified an abnormal vessel arising from the posterior aspect of the descending thoracic aorta at T7-T8, which extended superiorly in a tortuous manner along the posterior mediastinum, giving off several branches into the epidural plexus and eventually connecting to the left innominate vein (Figure 3, Video 2). There was an additional connection from this aberrant aortic vessel to the azygos vein.

**Figure 4:**
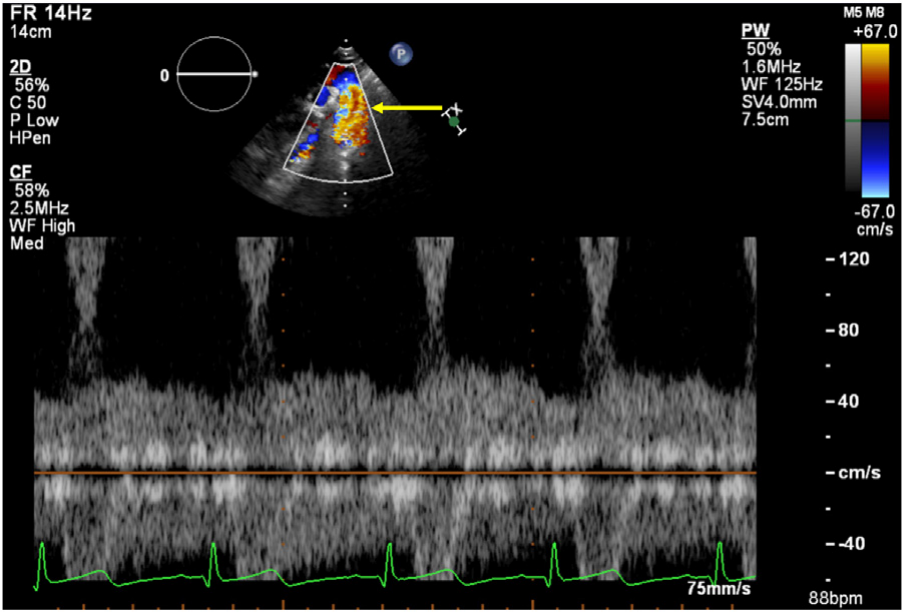
Two-dimensional TTE, suprasternal, sagittal view with color flow–guided continuous-wave Doppler, cursor within the descending aorta *(arrow)*, demonstrates cranially directed flow that may be misinterpreted as aortic regurgitation. However, given the continuous spectral Doppler flow pattern, this likely represents flow from a separate vessel in close proximity to the aorta rather than flow within the aorta itself.

**Figure 5:**
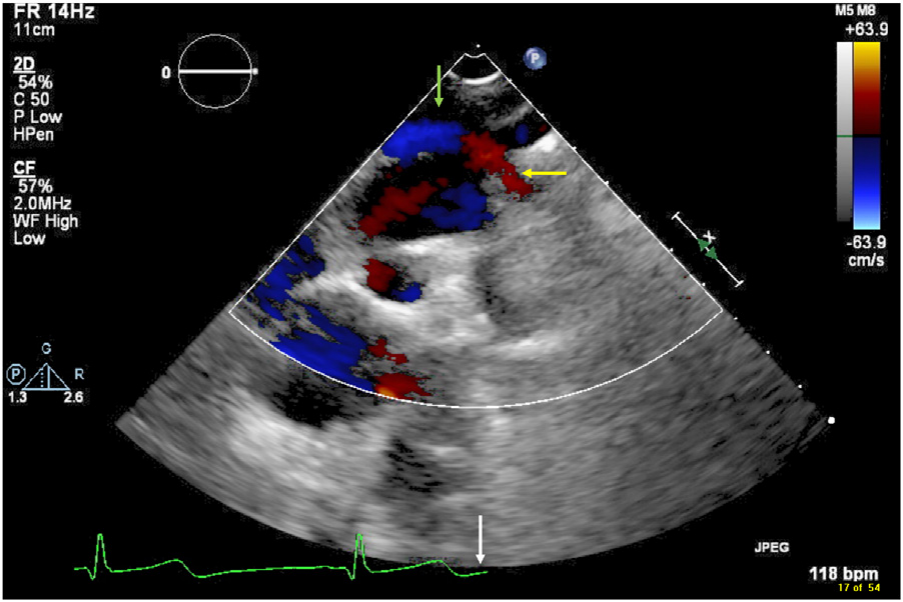
Two-dimensional TTE with color flow Doppler, suprasternal sagittal view, diastolic phase *(white arrow)*, demonstrates mild dilation of the innominate vein *(green arrow)* with red color flow suggesting an anomalous vessel *(yellow arrow)* coursing cephalad to enter the dilated innominate vein.

The abnormal chest CTA findings prompted retrospective review of the TTE. Video 3 and Figure 4 use the TTE sagittal suprasternal notch view to demonstrate the ascending anomalous vessel in close proximity to the aorta with color and spectral Doppler. Video 4 and Figure 5 demonstrate mild dilation of the innominate vein with color flow Doppler, suggesting an additional vessel (likely the AVF) coursing cephalad to enter the dilated innominate vein.

Since the patient presented asymptomatically and without chamber dilation, we elected for observation without intervention. At an office visit 17 months after initial presentation, the patient remained healthy and asymptomatic.

## Discussion

The differential diagnosis for a continuous murmur that radiates to the back includes patent ductus arteriosus, aortopulmonary window, arteriovenous or coronary fistula, ruptured sinus of Valsalva, and mammary souffle.^1^^,^^2^

Congenital systemic AVFs in the thorax are extremely rare. The first was reported in 1981 and was between the descending aorta, azygos vein, and superior vena cava.^3^ Subsequent cases have demonstrated thoracic AVF with arterial origins including aortic arch, thoracic aorta, intercostal arteries, and brachiocephalic truncus and venous connections including left innominate vein, hemiazygos vein, and superior vena cava.^4^, ^5^, ^6^, ^7^, ^8^

Thoracic AVF are either acquired—secondary to trauma or ruptured dissection or in patients with connective tissue disorders—or congenital.^1^ Congenital causes are rare and result from abnormalities in embryonic development. During development, venous blood with aortic origin above the third or fourth intercostal arteries drains into the left innominate vein and venous blood below these arteries drains into the azygos or hemiazygos veins.^1^^,^^3^ The abnormal vessel in our case originated from the level of T7-T8 and ultimately connected to the left innominate vein. Although there was an additional connection to the azygos vein, it is abnormal embryologically for a vessel originating below the fourth intercostal space to ultimately connect with the left innominate vein, and such a situation has been described only once previously.^1^

Most AVFs present with a continuous murmur audible with anterior chest wall auscultation with radiation to the back. A feature unique to this case is the fact that the murmur was only audible with posterior thoracic wall auscultation, therefore highlighting the importance of a thorough physical exam in all patients in the primary care setting. This patient was initially referred to the pediatric cardiology clinic due to the pediatrician’s careful physical examination. Sequelae of this AVF can include hemodynamically significant left-to-right shunt resulting in steal of spinal nerve arterial circulation; however, our patient demonstrated no neurologic deficits. The patient also had normal chamber sizes on echocardiography. While it would be expected to have a large left-to-right shunt based on the pressure difference between the arterial and venous beds connected by this anomalous vessel, the length and tortuosity of the vessel may be providing protective resistance and limiting flow. This theory is corroborated by the lack of right-sided chamber dilation on TTE and the absence of retrograde flow with abdominal aorta Doppler (Figure 2).

Given the rarity of thoracic AVF, there is little current evidence to guide management. Case reports demonstrate success with either surgical repair or transcatheter embolization.^9^ There is a case of spontaneous resolution of thoracic AVF after diagnostic catheterization, but this was thought to be secondary to irritation of the vessel during catheterization.^10^ Uysal and colleagues^7^ described a case where an AVF in an asymptomatic patient recurred after ligation. A review of current case reports for congenital systemic AVF demonstrates that most are intervened upon, either surgically or via cardiac catheterization, although more commonly when patients present with symptoms of congestive heart failure.^1^^,^^5^^,^^6^^,^^9^ In discussion with the family, it was determined that the risk associated with catheter-based interventions outweighed the benefit in an asymptomatic healthy child. As a result, our patient was managed conservatively with no intervention and yearly TTE to assess chamber sizes as sequelae of a significant shunt. Our patient is currently very active without limitations, but if this were to change, exercise testing may be helpful. If our patient were to become symptomatic, we would opt for percutaneous device placement for vessel occlusion.

## Conclusion

Aorta-innominate vein fistulous connection is a rare congenital anomaly. Patients may present with a continuous murmur in the absence of clinical symptoms. If there are no signs of arterial steal, heart failure, and/or significant left-to-right shunt, we would advocate for conservative management. If intervention is not performed, longitudinal follow-up should be arranged.

## Ethics Statement

The authors declare that the work described has been carried out in accordance with The Code of Ethics of the World Medical Association (Declaration of Helsinki) for experiments involving humans.

## Consent Statement

The authors declare that since this was a non-interventional, retrospective, observational study utilizing de-identified data, informed consent was not required from the patient under an IRB exemption status.

## Funding Statement

The authors declare that funding for this report was provided by NIH-Vanderbilt Developmental Determination of Cardiovascular Diseases, T32 Training Grant for Research Fellows (T32-5T32HL105334-10).

## Disclosure Statement

The authors report no conflict of interest.

## Supplementary Data

Video 1 Two-dimensional TTE (5 MHz transducer), apex-down (anatomically correct, pediatric) apical 4-chamber view, demonstrates normal biventricular chamber dimensions and function.

Video 2 Chest CTA, **t**hree-dimensional volume-rendered reconstruction, cine-rotational display, demonstrates an abnormal vessel arising from the posterior aspect of the descending thoracic aorta and extending superiorly in a tortuous manner along the posterior mediastinum. This vessel provides several branches to the epidural plexus and eventually connects to the left innominate vein.

Video 3 Two-dimensional TTE, suprasternal, sagittal view without *(left)* and with *(right)* color flow Doppler, demonstrates red color flow in the area of the distal transverse arch and descending aorta that represents a vessel coursing cephalad in a plane similar to the descending aorta. This color flow pattern was absent in a neutral position within the suprasternal notch but was accentuated with a slight tilt of the probe to the patient’s left consistent with the location of the anomalous vessel.

Video 4 Two-dimensional TTE with color flow Doppler, suprasternal sagittal view, demonstrates mild dilation of the innominate vein with red color flow suggesting a vessel coursing cephalad to enter the dilated innominate vein.
